# Induced pluripotent stem cells from subjects with Lesch-Nyhan disease

**DOI:** 10.1038/s41598-021-87955-9

**Published:** 2021-04-19

**Authors:** Diane J. Sutcliffe, Ashok R. Dinasarapu, Jasper E. Visser, Joery den Hoed, Fatemeh Seifar, Piyush Joshi, Irene Ceballos-Picot, Tejas Sardar, Ellen J. Hess, Yan V. Sun, Zhexing Wen, Michael E. Zwick, H. A. Jinnah

**Affiliations:** 1 Department of Neurology, Emory University School of Medicine, 101 Woodruff Circle, 6305 Woodruff Memorial Building, Atlanta, GA 30322 USA; 2 Department of Human Genetics, Emory University School of Medicine, Atlanta, GA 30322 USA; 3 Department of Neurology, Cognition and Behavior, Donders Institute for Brain, Radboud University Medical Center, Nijmegen, The Netherlands; 4 Department of Neurology, Amphia Hospital, Breda, The Netherlands; 5 Neurosciences Graduate Program, Graduate Division of Biological and Biomedical Sciences, Laney Graduate School, Emory University, Atlanta, 30322 USA; 6 Laboratoire de Biochimie Métabolomique Et Protéomique, Hôpital Universitaire Necker, Paris, France; 7 Department of Pharmacology and Chemical Biology, Emory University School of Medicine, Atlanta, GA 30322 USA; 8 Department of Epidemiology, Emory University Rollins School of Public Health, Atlanta, GA. 30322 USA; 9 Department of Psychiatry & Behavioral Sciences, Emory University School of Medicine, Atlanta, GA 30322 USA; 10 Department of Cell Biology, Emory University School of Medicine, Atlanta, GA 30322 USA; 11 Department of Pediatrics, Emory University School of Medicine, Atlanta, GA 30322 USA

**Keywords:** Induced pluripotent stem cells, Genetics research

## Abstract

Lesch-Nyhan disease (LND) is an inherited disorder caused by pathogenic variants in the *HPRT1* gene, which encodes the purine recycling enzyme hypoxanthine–guanine phosphoribosyltransferase (HGprt). We generated 6 induced pluripotent stem cell (iPSC) lines from 3 individuals with LND, along with 6 control lines from 3 normal individuals. All 12 lines had the characteristics of pluripotent stem cells, as assessed by immunostaining for pluripotency markers, expression of pluripotency genes, and differentiation into the 3 primary germ cell layers. Gene expression profiling with RNAseq demonstrated significant heterogeneity among the lines. Despite this heterogeneity, several anticipated abnormalities were readily detectable across all LND lines, including reduced *HPRT1* mRNA. Several unexpected abnormalities were also consistently detectable across the LND lines, including decreases in *FAR2P1* and increases in *RNF39*. Shotgun proteomics also demonstrated several expected abnormalities in the LND lines, such as absence of HGprt protein. The proteomics study also revealed several unexpected abnormalities across the LND lines, including increases in GNAO1 decreases in NSE4A. There was a good but partial correlation between abnormalities revealed by the RNAseq and proteomics methods. Finally, functional studies demonstrated LND lines had no HGprt enzyme activity and resistance to the toxic pro-drug 6-thioguanine. Intracellular purines in the LND lines were normal, but they did not recycle hypoxanthine. These cells provide a novel resource to reveal insights into the relevance of heterogeneity among iPSC lines and applications for modeling LND.

## Introduction

Lesch-Nyhan disease (LND) is an inherited disorder caused by pathogenic variants in the *HPRT1* gene^1^. The variants result in deficiency of a purine recycling enzyme, hypoxanthine–guanine phosphoribosyltransferase (HGprt)^2,3^. This enzyme is ubiquitously expressed in all tissues of the body at all stages of development^4^. HGprt deficiency produces pleiotropic clinical effects including overproduction of uric acid leading to kidney stones and gout^5^, brain abnormalities^6–8^, and macrocytic anemia^9^.

Many experimental models have been developed for LND. Animal models have been produced by knocking out the *Hprt1* gene in mice^10,11^, rats^12^ and rabbits^13^. Several studies have described spontaneous or induced mutations in immortalized rodent cell lines^14–20^. However, rodents do not exhibit the clinical features seen in affected humans^21,22^. One of the reasons for the lack of overt abnormalities in rodents may be that purine metabolism differs across species. For example, purine turnover is at least tenfold higher in rodents compared to primates. Rodents also express certain enzymes that are lacking in humans, such as uricase, which protects rodents from excess uric acid^23–25^. There are also substantial differences in organ development, particularly for the brain. Thus, species differences in purine metabolism or neurodevelopmental mechanisms have led to uncertainty regarding the translation of findings from rodent models to humans.

Other experimental models have been derived from human materials, such as blood cells^26–32^ or fibroblasts^3,33–38^ collected from individuals with LND. Some cell models have relied on spontaneous or induced mutations in immortalized human cell lines^39–42^. One study described human neural stem cells from an aborted LND fetus^43^, while another focused on partial knockdown of *HPRT1* expression in a human induced pluripotent stem cell (iPSC) line and a human embryonic stem cell line^44^. These studies of human cells have revealed that many of the consequences of HGprt deficiency are cell-specific^42,45^. Three reasons account for these cell type-specific differences. First, HGprt is expressed ubiquitously; but its level varies across tissues, suggesting that some cells are more dependent on HGprt than others^46^. Other enzymes of purine metabolism also are expressed at different levels in different tissues^47^, so downstream and compensatory consequences of HGprt deficiency may vary as well. Finally, purines are utilized in different ways by different tissues. For example, rapidly dividing cells use purine nucleotides predominantly for RNA synthesis and DNA replication^48^. In contrast, post-mitotic cells such as neurons do not need large amounts of purine nucleotides for DNA replication, but use nucleotides and nucleosides for signaling both within and between cells^49^.

Because the consequences of HGprt deficiency vary among different types of cells, the pathogenesis of the diferent clinical aspects of HGprt deficiency may need to be studied in different cell types relevant to the different clinical features of LND. Induced pluripotent stem cells (iPSCs) derived from patients carrying pathogenic variants in the *HPRT1* gene provide a novel strategy to study the consequences of HGprt deficiency in different lineages. The main goal of the current study was to describe the development and characterization of 6 iPSC lines derived from 3 unrelated LND cases as a resource for future studies. The first part of the manuscript describes the generation and characterization of the novel iPSC lines. The second part describes gene and protein expression abnormalities consistently found among the LND lines. The final part presents a functional characterization of the LND lines in relation to purine metabolism.

## Results

### Selection of fibroblasts for reprogramming

From a collection of fibroblast cultures of 47 cases of HGprt deficiency, several criteria were used to select candidates for reprogramming. First, the mutation should be stable with a known consequence for HGprt enzyme function. Second, the mutation should be associated with multiple unrelated LND patients to ensure a consistent relationship with the clinical phenotype. Third, cultures from relatively young patients should be available, because age affects reprogramming efficiency and frequency of acquired genetic variants^50^. With these criteria in mind, three unrelated cases were selected, all with the classic LND phenotype that included overproduction of uric acid, motor and cognitive disability, and self-injurious behavior. Two involved hot-spot variants leading to different stop codons (c.151C>T and c.508C>T) and one was frame-shifting mutation (c.371insTT). All of these variants lead to low mRNA levels due to nonsense-mediated decay, as well as null enzyme function. Control fibroblast cultures were selected from a bank of 20 normal individuals free of any known medical or neurological problems. Because LND is inherited in an X-linked recessive manner, all cases and controls were males (Table 1).

**Table 1:** Subject characteristics.

	LND Cases			Healthy controls		
	A	D	T	A	B	C
Sex	Male	Male	Male	Male	Male	Male
Age^a^	15	10	46	21	43	22
Race	Black	Caucasian	Caucasian	Caucasian	Black	Caucasian
*HPRT1* gene variant	c.151C > T	c.508C > T	c.371insTT	none	none	none
HGprt consequence	Premature stop	Premature stop	Frame shift, premature stop	Normal	Normal	Normal
Clinical features^b^	Classic	Classic	Classic	Normal	Normal	Normal
iPSC lines	L3.29 L3.30	L2.09 L2.12	L1.02 L1.04	C1.02 C1.03	C2.04 C2.06	C3.05 C3.08

^a^Age relates to subject age at skin biopsy.

^b^Clinical features of classic LND include motor dysfunction resembling cerebral palsy, cognitive impairment, self-injurious behavior, and overproduction of uric acid.

### Characterization of iPSC lines

**Figure 1:**
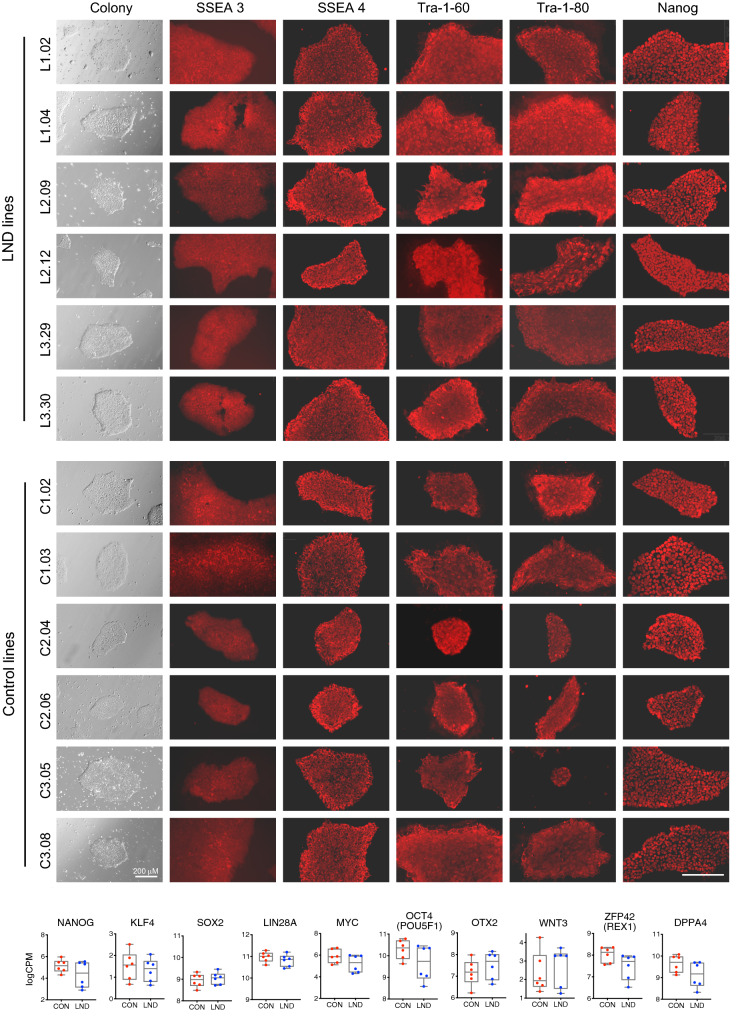
Pluripotency characteristics. This figure shows a phase-contrast photomicrograph of typical colonies for all 6 LND and all 6 control lines, along with immunostains for pluripotency markers (SSEA3, SSEA4, TRA-1-60, TRA-1-80, Nanog). All cultures were stained simultaneously, and all photomicrographs were taken using the same microscope settings. Scale bar is shown at bottom right and depicts 200 μM. Gene expression profiling shown as box plots at the bottom of the figure reveal that the control and LND lines expressed similar levels of genes associated with pluripotency.

**Figure 2:**
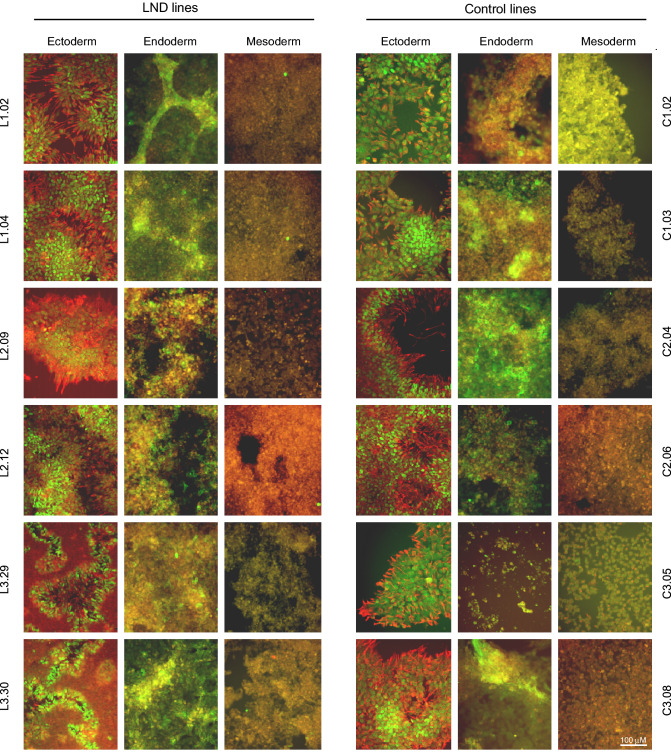
Trilineage differentiation. This figure shows the differentiation of all 6 LND and all 6 control lines into the 3 primary germ cell layers via merged immunostains for ectoderm (PAX6 = green, nestin = red), mesoderm (Brachyury = green, NCAM = red), and endoderm (SOX17 = green, FOXA2 = red). All cultures were stained simultaneously, and all photomicrographs were taken using the same microscope settings. Distinct red or green colors are evident for ectoderm, where the markers were often expressed in different cells. Shades of orange and yellow result from varied proportions of overlapping red and green markers. Scale bar is shown at bottom right and depicts 100 μM.

At the morphological level, all 6 LND and all 6 control lines grew in rounded colonies with defined borders typical of other iPSC lines (Fig. 1). All showed immunostaining characteristics typical of pluripotency including expression of SSEA3, SSEA4, TRA-1-60, TRA-1-80, and Nanog (Fig. 1). Complete gene expression profiles were determined by RNAseq, with the percentage of mapped reads ranging between 88.8 and 91.7%. A total of 23,748 genes were detected. After filtering genes expressed at very low levels, 17,113 genes remained for quantification. All of the lines expressed genes typically associated with pluripotency, with no apparent differences between LND and control lines (Fig. 1). All lines were capable of differentiating into each of the three main germ cell layers as shown by immunostains (Fig. 2) for ectoderm (PAX6, NESTIN), mesoderm (Brachyury, NCAM), or endoderm (SOX17, FOXA2). Over several different staining runs, with or without a nuclear counterstain, there were no obvious differences between the LND and control lines.

RNAseq data for all iPSC lines were queried to assess for genetic variants in *HPRT1*. Each of the LND lines harbored genetic variants expected from their starting fibroblast lines (Table 1, Supplementary Fig. S1). The nonsense variant c.151C>T was confirmed for lines LND29 and LND30. The nonsense variant c.508C>T was confirmed for lines LND9 and LND12. The frame-shifting variant c.371insTT was confirmed for LND2 and LND4. All of these variants were independently confirmed by sequencing RT-PCR products. No other *HPRT1* sequence variants were detected in the LND iPSC lines and no variants were detected in any of the 6 control iPSC lines. Karyotypes were normal for all LND and control lines, except for line LND30 where 11/20 metaphases demonstrated an unbalanced translocation affecting the long arm of chromosome 1, with a karyotypic profile of 46,XY^51^/46,XY,t(1:10)(p36.3;q11.1)^51^ (Supplementary Fig. S2). Although karyotypically abnormal lines may yield idiosyncratic results, this line was not discarded, but results were censored so that it could be compared against the other karyotypically normal lines.

### Differential gene expression in LND and control iPSCs

**Figure 3:**
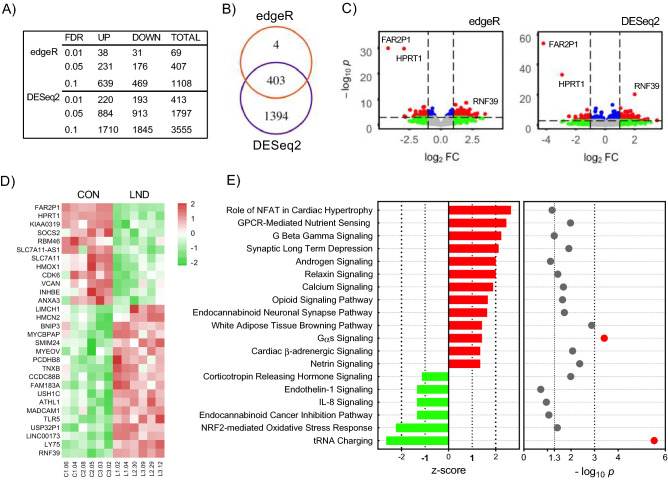
Transcriptome analysis. Gene expression profiles were evaluated by RNAseq for all LND (n = 6) and control (n = 6) lines. Differential gene expression differed according to both analytical method (edgeR, DESeq2) and statistical threshold (**A**). However, 403 genes were differentially expressed using both methods at FDR < 0.05 (**B**). For both edgeR and DESeq2 methods, volcano plots (**C**) consistently demonstrate 2 genes to be expressed at markedly lower levels in the LND lines (*HPRT1* and *FAR2P1*), and 1 gene to be expressed at higher levels in the LND lines (*RNF39*). The red color shows genes with FDR < 0.05 and fold change > 2. The blue color shows genes with FDR < 0.05 and fold change < 2. Green and gray show gene changes with FDR > 0.05. Individual variation among the lines is shown as a heatmap of gene expression (**D**). Red depicts higher gene expression and green depicts lower expression. Considering only the 403 genes differentially expressed using both edgeR and DESeq2 at FDR < 0.05, 19 pathways appeared to be significantly different when comparing the LND and control lines (**E**). Red bars depict positive z-scores while green depict negative z-scores. Red dots represent p-values < 0.001 (i.e. − log10(.001) > 3.

The RNAseq data were next evaluated to identify differences in gene expression between the LND (n = 6) and control (n = 6) lines. With a minimum threshold of 1.5 × change and correcting for multiple comparisons with FDR method, differential gene expression using DESeq2 method revealed significant differences for 3555 genes at FDR < 0.10, 1797 transcripts at FDR < 0.05, and 413 transcripts at FDR < 0.01 (Fig. 3A). Differential gene expression using the edgeR method revealed significant differences for 1108 transcripts at FDR < 0.10, 407 transcripts at FDR < 0.05, and 69 transcripts at FDR < 0.01 (Fig. 3A). These results demonstrate that these commonly used methods for differential gene expression yield different outcomes. However, at FDR < 0.05, almost all transcripts (403 transcripts, 99%) were common to both the DESeq2 and edgeR methods (Fig. 3B). As shown in volcano plots (Fig. 3C) and heat map (Fig. 3D), the most prominently increased transcript was *RNF39* (ring finger protein 39). The most prominently reduced transcripts were *HPRT1* and *FAR2P1* (fatty acyl-coA reductase pseudogene 1). All three of these prominently altered transcripts in RNAseq were confirmed via qPCR using independently collected samples from each line (Supplementary Fig. S3). The significant decrement in *HPRT1* transcripts provides a useful positive control, because such a reduction is consistent with the fact that the genetic variants included here are all subject to nonsense-mediated mRNA decay.

Genes that were differentially expressed via either the edgeR and DESeq2 methods were next subject to Ingenuity Pathway Analysis. Although these methods revealed somewhat different lists of differentially expressed transcripts, they produced similar results for affected biological pathways (Fig. 3E). Of the top 10 pathways selected based on p-value and z-scores, two were significantly reduced (tRNA charging and NRF2-mediated oxidative stress) and 8 were significantly increased (Role of NFAT in cardiac hypertrophy, GPCR-mediated nutrient sensing in enteroendocrine cells, androgen signaling, synaptic long-term depression, endocannabinoid neuronal synapse pathway, G beta gamma signaling, calcium signaling, and relaxin signaling).

### Shotgun proteomics in LND and control iPSCs

An unbiased shotgun approach was used to compare the proteomes of the LND (n = 6) and control (n = 6) iPSC lines. After filtering peptides with low expression, a total of 33,648 peptides mapping to 2,493 proteins were quantified. The proteomics dataset provided quantitative data for 12 proteins associated with pluripotency (ACACA, ACLY, CDH1, DNMT1, DNMT3A, FASN, RBM3, SOX2, SLC25A1, VIM, YAP1, ZIC2). None of these were significantly different between LND and control iPSCs (not shown).

**Figure 4:**
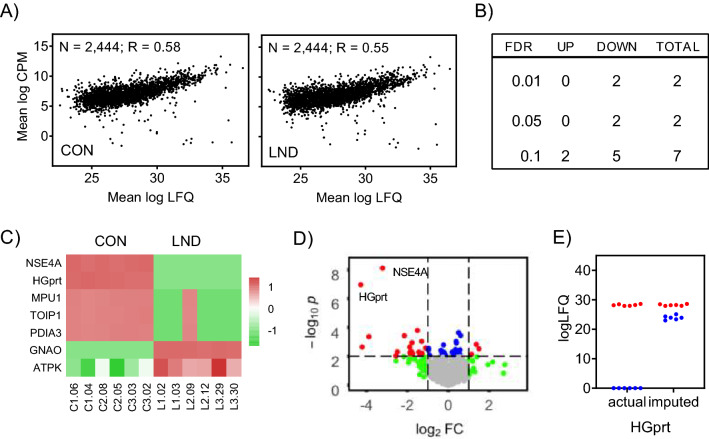
Proteomics analysis. Protein expression profiles were evaluated by shotgun proteomics for all LND (n = 6) and control (n = 6) lines. There was a good quantitative correlation between the gene and protein expression results for both the LND and control lines (**A**). Only 7 proteins reached statistical thresholds for differential expression at FDR < 0.10 (**B**). Individual variation for among the lines for the 7 proteins meeting the FDR < 0.10 criterion is shown as a heatmap (**C**). Red depicts a higher gene expression and green depicts lower gene expression. Only 2 proteins remained significantly different between LND and control at FDR < 0.01, and a volcano plot revealed marked reductions in the levels of both HGprt and NSE4A (**D**). Red depicts genes with p < 0.001 and fold change > 2. Green and gray depict FDR > 0.05. The marked reductions in all LND lines (blue) compared to all control lines (red) were confirmed by evaluation of the raw and imputed values for HGprt (**E**).

The proteomics data were next evaluated to identify differences in protein expression between the LND and control lines. There were 7 significant protein changes at FDR < 0.10 (Fig. 4B). Five proteins were decreased (HGprt, NSE4A/EID3, MPU1, TOIP1, PDIA2, GNAO, PDIA3), and 2 proteins were increased (GNA0, ATPK) (Fig. 4C). Two of these protein changes remained significant at FDR < 0.01 (Fig. 4D). One was HGprt, as expected for the types of mutations involved. In fact, comparison of the raw vs imputed proteomics data implied a complete absence of HGprt protein in all of the LND samples (Fig. 4E). The other peptide significantly reduced at FDR < 0.01 mapped to both NSE4A (non-structural maintenance of chromosomes element 4, homolog A) and EID3 (EP300 interacting inhibitor of differentiation 3, a paralog of NSE4A). Both the raw and imputed data for NSE4A/EID3 implied a complete absence in all LND iPSCs. Reductions in HGprt and NSE4A were confirmed using Western blotting in independently collected samples from all the lines (Supplementary Fig. 4).

### Comparison of RNAseq and proteomics results

Among 2493 proteins identified, 2444 mapped to genes simultaneously quantified by RNAseq. Overall, there was a good quantitative correlation between the results from proteomics and RNAseq (Fig. 4A) for both the control (R = 0.58) and LND lines (R = 0.55). These correlations fall in the range of other studies that compared RNAseq and proteomic data generated from the same samples^51^. The lack of strict correlation between these methods is known to result from post-transcriptional, post-translational, and other mechanisms not detected by either method.

Among the analytes that were most significantly different between control and LND lines, RNAseq and proteomics revealed significantly reduced *HPRT1* transcripts and HGprt protein. However some of the transcripts significantly different between LND and controls in the RNAseq dataset were not significantly different in the proteomics study. For example *FAR2P1* is a pseudogene, so no protein was detected by proteomics. *RNF39* was significantly increased in the RNAseq dataset, but was not detectable in the proteomics dataset. Thus some of the apparent discrepancies in the RNAseq and proteomics datasets reflect differences in the biology of mRNA and protein expression.

Conversely, some statistically significant differences in the proteomics dataset between control and LND iPSCs were not significant in the RNAseq dataset. For example, a peptide mapping to both EID3 and NSMCE4 was detected in control iPSCs, but absent in LND iPSCs. However, the RNAseq revealed no significant change in either transcript (*NSMCE4*, p = 0.45; *EID3*, p = 0.23). Two other proteins significantly altered in the proteomics study also were not significantly altered by RNAseq. These proteins included ATPK (gene symbol *ATP5JK* or *ATP5MF*, p = 0.11) and GNAO (gene symbol, *GNAO1*, p = 0.24). ATPK is involved in synthesis of the purine ATP and GNAO is regulated by the purine GTP, so changes at the protein level may reflect post-transcriptional alterations caused by changes in intracellular purine metabolism in HGprt-deficient cells. Three other proteins significantly altered in the proteomics study were not significantly altered in the RNAseq study when statistically correcting for multiple comparisons, but they showed clearly significant trends in the same direction as proteomics when uncorrected. These proteins included PDIA3 (gene symbol *PDIA3*, p = 0.010), MPU1 (gene symbol *MPU1*, p = 0.011), TOIP1 (gene symbol, *TOR1AIP1*, p = 0.025). Thus some of the apparent discrepancies between the RNAseq and proteomics datasets reflect the limits of statistical methods in RNAseq and proteomics, and some have argued that corrections for multiple comparisons using the FDR method are inappropriately stringent for proteomics^52^. These comparisons highlight the many different reasons that RNAseq and proteomics datasets from the same samples typically show imperfect correlations ranging from 0.40 < R < 0.60^51^.

### Functional assessment of LND iPSCs

**Figure 5:**
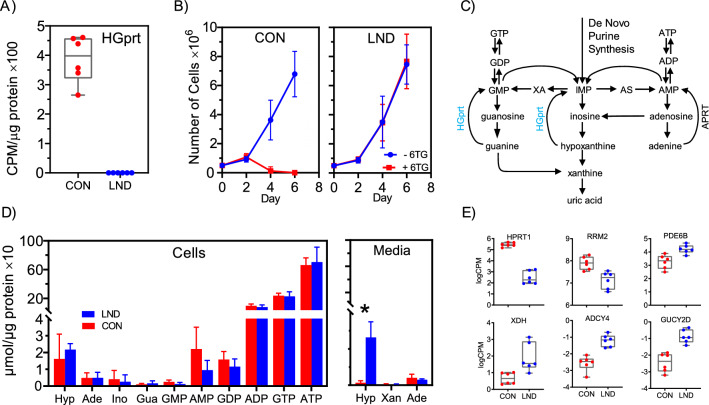
Functional assessment of HGprt deficiency. All control iPSC lines (red, n = 6) demonstrated readily detectable activity in the HGprt enzyme assay, while all LND iPSC lines (blue, n = 6) had no detectable activity (**A**). Results are shown as box plots with results for each line shown separately. There was no obvious difference in the growth of the control and LND lines in the absence of 6TG (blue lines, **B**). However, the control lines showed a clear toxic effect of 6TG, while the LND lines appeared to be resistant to it (red lines, **B**). In these growth curves, the error bars show the standard deviation. HGprt plays an important role in the metabolism of hypoxanthine (**C**), leading to marked hypoxanthine accumulation that is released into the tissue culture medium; with no apparent impact on intracellular purines (**D**). In this panel, controls are shown in red and LND in blue, with error bars reflecting standard deviations. Among 109 genes in the KEGG pathway for purine metabolism, only 6 were significantly different between control (red) and LND (blue) iPSC lines at FDR < 0.05 (**E**).

To assess the functional effect of the *HPRT1* genetic variants, all 6 LND and all 6 control lines were evaluated using an assay for HGprt enzyme activity. HGprt enzyme activity was readily detectable in all control iPSCs (Fig. 5A). Functional HGprt enzyme activity was absent from all LND lines, consistent with the transcriptome and proteome results described above. To further assess the impact of the *HPRT1* genetic variants on cell growth, cells were grown with or without 6TG, an inert synthetic purine base that is phosphorylated into a toxic purine nucleotide, only in cells which have functional HGprt. Overall, the average growth rates for the LND and control iPSCs were comparable in the standard culture medium (Fig. 5B). All of the control lines showed a clear toxic effect when 6TG was added, while all LND were resistant to it (Fig. 5B). These results demonstrate absence of functional HGprt enzyme in all of the LND iPSC lines.

Because HGprt plays a known role in purine salvage (Fig. 5C), purines were measured in all 6 LND and all 6 control iPSCs by HPLC with photodiode array UV detection. In keeping with other types of cells, the majority of intracellular purines were adenine nucleotides (ATP, ADP, AMP), with smaller amounts of guanine nucleotides (GTP, GDP, GMP), and trace amounts of several other purines. Multivariate ANOVA revealed no significant differences for any of the intracellular purines when comparing the LND vs control lines (Fig. 5D).

Purines accumulated in the tissue culture medium over a 24-h period were also quantified because some purines are released from cells to the extracellular environment. In keeping with other types of cells, the tissue culture medium contained purine bases and nucleosides, but no detectable nucleotides. Multivariate ANOVA revealed significant differences between the LND and control lines for hypoxanthine (Fig. 5D). The LND iPSCs released an average of 20 fold more hypoxanthine into the medium than control iPSCs. This result is consistent with the known role of HGprt in recycling hypoxanthine into purine nucleotides. Uric acid levels were undetectable in both LND and control iPSCs, implying that the oxidase form of xanthine oxidoreductase (XOR) is not active in these cells (Fig. 5D).

Although HGprt deficiency is the primary biochemical defect in LND, it is known to produce numerous downstream secondary changes in purine metabolism, such as increases in purine synthesis. We therefore interrogated the RNAseq data for changes the expression of other genes encoding proteins known to be involved in purine metabolism. Among the 109 purine-related genes in the KEGG pathway for purine metabolism, only 6 were significantly different in the LND vs control iPSC lines at FDR < 0.05 (Fig. 5E). This result implies that any secondary changes in purine metabolism that occur in LND, such as increases in purine synthesis, may result from post-transcriptional mechanisms. This suggestion is consistent with prior studies have shown that many enzymes of purine metabolism not regulated by their levels of expression, but by other factors such as substrate availability, release from endogenous feedback inhibitors, post-translational mechanisms, or compartmentalization into purinosomes^48,53^.

## Discussion

The current studies describe the development and characterization of 6 iPSC lines from subjects with classic LND, along with 6 iPSC lines from normal healthy individuals for comparisons. All 12 lines have the typical characteristics of pluripotent stem cells, as assessed using multiple methods including immunostaining for pluripotency markers, expression of mRNA transcripts for pluripotency genes, and directed differentiation into the 3 primary germ cell layers (endoderm, ectoderm, and mesoderm). These cells provide novel tools for studying the pathogenesis of LND, as well as novel insights into the biology of iPSCs and their use for disease modeling.

### Relevance for iPSC biology

Many prior studies have shown an important interplay between pluripotency and metabolism, and especially purine nucleotides such as ATP^54^. In addition to driving many energy-dependent reactions, ATP is required as a building block for DNA and RNA in dividing cells. Mammalian cells derive their purine nucleotides from two major sources, de novo purine synthesis and purine salvage^48^. However, all types of cells do not rely equally on de novo and salvage pathways to maintain purine levels. For example, actively dividing stem or neoplastic cells typically rely heavily on de novo synthesis, while terminally differentiated cells tend to depend more on salvage^48,55–57^.

Although *HPRT1* is listed among the many genes involved in cell cycling^58^, our studies imply that iPSCs do not require HGprt, because iPSCs with null variants had pluripotency characteristics that were similar to those of iPSCs derived from normal individuals (Figs. 1, 2), they grew at rates similar to iPSCs from normal individuals (Fig. 5B), and they demonstrated no significant changes in intracellular purines (Fig. 5D). However, all 6 LND iPSCs consistently wasted large amounts of purines, as judged by the 20-fold increases in hypoxanthine released from cells into the tissue culture medium (Fig. 5D). Because intracellular purine nucleotides were normal in the LND iPSCs, this result implies that the de novo pathway for synthesis of new purines is sufficient to compensate for any purines potentially lost because of the absence of HGprt-mediated purine salvage in iPSC cells.

### Relevance for iPSC disease modeling

Many prior studies of iPSCs derived from subjects with various diseases have relied on relatively small numbers of iPSC lines, especially for rare diseases^59^. For LND, prior studies have described only 1–2 iPSC lines, often from a single individual^60,61^. Others have described *HPRT1* gene-edited iPSCs^62^. However, few characteristics regarding these iPSCs were provided. Many other studies have emphasized phenotypic variation among iPSC lines, even those derived from the same subject^63–70^. There are many causes for this variability including sample source (disease characteristics, type of mutations, background genome, sex, age, race, etc.…), cell handling methods (reprogramming method, growth conditions, passage level, etc.….), and experimental measures examined (gene expression, protein expression, enzyme activity, purines measures, etc.…). Our studies with 6 LND iPSC lines (2 independent lines from each of 3 unrelated subjects) provide some insights into the biological relevance of this variability.

On the one hand, our studies confirm significant variation between iPSC lines. For example, the L3.30 line acquired a genomic defect involving an unbalanced translocation of chromosome 1. Such chromosomal defects are commonly acquired during iPSC production and growth^71,72^. Significant variability was also evident across iPSC lines in the gene expression studies, with no obvious influence of within-subject or between-subject relationships (Fig. 3D). In the proteomic studies, line L2.09 demonstrated idiosyncratic results compared to other LND lines (Fig. 4C). This variability evident across the 6 LND iPSC lines confirms prior calls for caution when relying on a small number of iPSC lines for disease modeling, and calls into question results from prior smaller studies of LND^60,61^.

On the other hand, despite this variation, some very consistent abnormalities could be detected across the LND iPSC lines. For example, it is often claimed that karyotypical defects such as those found in LND30 should not be used, because they provide idiosyncratic results^71,72^. However, our results show that this line behaved similarly to the other 5 LND iPSC lines in all respects studied. Thus this karyotypical defect did not seem relevant to the molecular and biochemical consequences of HGprt deficiency. Despite the variation in gene expression, consistent abnormalities were clearly detectable for many transcripts. Most notably, consistent abnormalities could be detected in the LND iPSC lines for expression of *HPRT1* mRNA and protein, enzyme activity, growth characteristics with and without 6TG, and certain unexpected findings such as reductions in *FAR2P1* mRNA (Fig. 3) and NSE4A/EID3 protein (Fig. 4). Thus, biological variation certainly exists among iPSCs, but the significance of this variation depends on what is being studied. Our results demonstrate that the most robust disease-related abnormalities can be detected despite the variability inherent across iPSC lines.

### Relevance for LND

The results described here also demonstrate the value of both hypothesis-driven and unbiased approaches to understanding the biology of disease. LND is caused by defects in a known gene encoding a known protein with a known function. This knowledge makes it possible to hypothesize and then verify findings from studies using iPSCs, such as reductions in *HPRT1* mRNA in gene expression studies, loss of HGprt protein in proteomic studies, absence of HGprt enzymic activity, resistance to 6-TG, and specific changes in purine metabolism.

The unbiased studies further revealed some novel and unexpected findings relating to the loss of HGprt, such as marked and consistent reductions in *FAR2P1* mRNA in the LND iPSCs (Fig. 3). The reasons for changes in *FAR2P1* are not immediately obvious. It is thought to be a pseudogene with no known function. Further, there is no obvious connection between *FAR2P1* and *HPRT1*, the known biology of HGprt, or purine metabolism. Similarly, there was a significant reduction in a peptide mapping to both NSEA4 and/or EID3 in proteomic studies of the LND iPSCs (Fig. 4). Once again, the reasons for these changes are not clear. NSEA4 is thought to play a role in maintaining chromosome structure, and EID3 plays a role in suppressing gene transcription from nuclear receptors. Neither protein bears any obvious relationship to the biology of HGprt. These findings and their relationship to the biology of LND will require further studies.

HGprt deficiency appears to have minimal impact on intracellular purines in iPSCs, possibly because they depend more on de novo purine synthesis, rather than purine recycling, for maintaining purines. There are significant variations in dependence of different cells on synthesis versus salvage pathways to maintain purines. Tissue differences are most obvious in bone marrow cells and particularly mature erythrocytes, which lack a functional de novo synthetic pathway because of the absence of the first enzyme in the pathway^45,46,73^. Indeed, the dependence of erythrocytes on HGprt-mediated salvage of hypoxanthine supplied into the circulation by the liver^74^ may explain why macrocytic anemia is so common in LND^9,45^. Similarly, de novo purine synthesis seems to contribute to cell division and neurogenesis in the developing brain^56,75–77^, but the mature brain depends more on purine salvage^55,56,78^. Within the brain, neurons seem to express much more HGprt than glia^79^. In keeping with the concept that the ratio of de novo synthesis to salvage synthesis of purines is low in the adult brain, the Human Protein Atlas indicates high levels of HGprt across almost all brain regions, with variable or low levels of the 6 enzymes in the de novo pathway (www.proteinatlas.org). However, the distribution and levels of each enzyme of purine synthesis in various types of neurons, and whether they can aggregate into purinosomes needed to drive purine synthesis, has never been methodically mapped out^47,80,81^. Although the exact mechanisms remain to be disclosed, the dependence of specific neurons on HGprt-mediated purine salvage to maintain purines may explain the prominent neurobehavioral abnormalities of LND^82,83^. Thus, certain cell types may experience significant purine deficiency in LND, even when the undifferentiated LND iPSCs do not. Hypotheses relating to cell-specific changes in purines can now be tested by differentiating the LND iPSCs into disease-relevant cell types.

## Methods

### Fibroblast cultures

All procedures involving human subjects followed appropriate institutional and national guidelines and were approved by the Emory University Institutional Review Board. For all control subjects who provided data or materials for these studies, informed written consent was obtained. For LND subjects who have substantial cognitive impairment and usually cannot hold a pen, informed written consent was obtained their legal guardians; verbal assent also was obtained from subjects who could speak. The diagnosis of LND was made according to published criteria and was based on the presence of all elements of the classical clinical phenotype including self-injurious behavior, motor impairments, intellectual disability, and evidence for overproduction of uric acid^6^. The diagnosis was verified by biochemical testing showing reduced HGprt enzyme activity in fibroblasts or blood cells and/or an associated genetic variant predicting null HGprt enzyme activity. Cultures of human skin fibroblasts were prepared as previously described^3^.

### Reprogramming methods to generate induced pluripotent stem cells

Fibroblasts were reprogrammed to iPSCs, using a microRNA/mRNA Reprogramming method (Stemgent, Cambridge MA). Two independent lines for each of 3 unrelated LND cases were prepared, for a total of 6 lines. Two independent lines for each of 3 unrelated normal controls (n = 6) were prepared in parallel. For each fibroblast culture, one well of a 6-well plate was seeded with 5.0 × 10^3^ cells. Twelve days of transfections with in vitro transcribed mRNAs encoding the human transcription factors Oct4, Sox2, Klf4, c-Myc, Lin28 and nGFP mRNA followed. In addition, transfections at day 1 and day 5 included a proprietary miRNA cocktail. Colonies began to emerge at day 13–15. Colonies with typical iPSC morphology were manually passaged approximately 4 times, before being enzymatically passaged using Accutase (Stemcell Technologies). Cultures were typically maintained on Matrigel-coated 6-well plates (BD Biosciences) in an atmosphere of 5% CO_2_ at 37˚C in mTeSR1 medium (Stemcell Technologies) supplemented with 0.5% penicillin–streptomycin. The majority of studies were conducted after 10 passages.

### Genetic evaluation of iPSCs

For each iPSC line, one Matrigel-coated T25 flask of cells was grown to 80% confluency, harvested, and trypsin G-banded. A minimum of 20 metaphase cells were evaluated for karyotypic abnormalities (Wicell Research Institute Cytogenetics Lab, Madison WI). At least 4 metaphases were photographed with a minimum of 400 band resolution.

The original *HPRT1* genetic variant was confirmed in the reprogrammed iPSCs by RT-PCR. RNA was extracted using TriZol Reagent (Life Technologies, Carlsbad CA) and the concentration of RNA was determined using a NanoDrop spectrophotometer 1000 (Thermo Fisher Scientific, Rockford IL). RT-PCR was performed using Superscript One-Step for Long Template Kit (Life Technologies, Carlsbad CA) with 1 µg RNA (forward primer GCGAACCTCTGGGCTTTC, reverse primer AAGCTCTACTAAGCAGATGGCCACAGAACTAGA). After assessing the quality of the product by gel electrophoresis, cDNA was purified using the QIAquick PCR purification Kit (Qiagen, Venlo, The Netherlands) and samples, premixed with the primers, were sequenced (Genewiz, South Plainfield NJ). The sequencing result was analyzed by aligning it to the sequence of *HPRT1* (NM_000194.2) using BLAST aligning tool.

### Assessment of pluripotency

Several methods were used to assess pluripotency potential including immunostaining for pluripotency markers (SSEA3, SSEA4, TRA1-60, TRA1-81, NANOG), assessing expression of pluripotency genes by gene expression profiling, and differentiating the lines towards each of the 3 major germ cell layers. Primary antibodies for staining included anti-Tra1-81 (mouse, R&D #MAB8495), Anti-Tra1-60 (mouse, R&D #MAB4770), Anti-NANOG (rabbit, R&D # AF1997), Anti-SSEA-3 (mouse, R&D #MAB1434) or Anti-SSEA-4 (rat, Abcam #ab16287). Secondary antibodies included anti-mouse Alexa Fluor 488 and Alexa Fluor 594, anti-rabbit Alexa Fluor 594, anti-rat Alexa Fluor 488 or anti-goat Alexa Fluor 488 (Life Technologies, Carlsbad CA) diluted 1:1000 in 5% BSA-PBS. Cells were visualized using an Olympus DP72 Microscope (Olympus, Shinjuku, Tokyo, Japan). All antibodies used had their staining characteristics verified using the previously validated KOLF-2 iPSC line^84^. All control and LND lines were subject to at least 2 separate staining runs for all markers, with or without a nuclear counterstain (DAPI) to identify unstained cells.

The STEMdiff Trilineage Differentiation Kit (Stemcell Technologies, Vancouver BC) was used to assess competence for differentiation into the 3 major germ cell layers (endoderm, ectoderm and mesoderm). For ectoderm, antibodies targeted PAX6 (rabbit, Abcam #ab195045) and Nestin (mouse, Stemcell Technologies #60,091). For mesoderm, antibodies targeted Brachyury (goat, R&D #AF2085) and NCAM (mouse, Stemcell Technologies #60,021). For endoderm, antibodies targeted SOX17 (goat, R&D Systems #AF1924) and FOXA2 (mouse, BD Pharmingen #561,580). All lines were subject to at least 2 separate staining runs for all markers, with or without a nuclear counterstain (DAPI).

### Gene expression profiling

Prior studies of the RNAseq method have suggested ideal comparisons should involve at least 6 biological replicates^85^. As a result, each of the 6 control and 6 LND lines were grown to approximately 80% confluency, harvested by enzymatic release and centrifugation. At least 1 million cells per tube were flash-frozen in a mixture of ethanol and dry ice, and stored at –80˚C. Once samples from all lines were ready for simultaneous processing, RNA was isolated with the Qiagen miRNEasy kit with on-column DNAse treatment. Variance from biological replicates is far greater than variance from technical replicates, so the value of technical replicates is limited^86^. As a result, RNAseq measures were conducted only once for each cell line. RNA concentrations were measured with UV absorbance on a Tecan Infinite m200 pro. RNA profiles were assessed with the Agilent 2100 Bioanalyzer and Agilent RNA 6000 Nano Chip assay. RNA sequencing (RNA-seq) was conducted at HudsonAlpha (Birmingham, Alabama). A total of 50 million paired-end, 100 bp reads were generated per library (100 million total reads per sample). Base calling and FASTQ generation were processed using Illumina’s HiSeq Control Software version 1.5.15.1 (RTA v1.13.18 and bclfastq v 1.8.3).

Raw Illumina sequencing reads were first checked by FastQC (0.11.4) for quality and then aligned to the human indexed reference genome (UCSC RefGene; hg38 build, 26,485 genes) using STAR (Spliced Transcripts Alignment to a Reference)^87^. Quantification of gene expression was done using HTSeq-count^88^. To improve the fidelity of quantitative results, transcripts with very low counts across all libraries were filtered out^89^. This filtering strategy applied using filterByExpr function (default settings) from the edgeR (3.28.0) package retained 17,113 genes for differential expression analyses. Differential expression was examined using both edgeR and DESeq2 (1.26.0)^86^. Raw count data from HTSeq-count were imported into R (version 3.6.1; R Core Team, 2019, “R: A language and environment for statistical computing”, R Foundation for Statistical Computing, Vienna, Austria. https://www.R-project.org/) where they were filtered, normalized and identified. For edgeR, counts per million (CPM) data were generated with the trimmed method of M-values (TMM), as implemented in the Bioconductor package edgeR^90^. CPM is defined as read counts scaled by the number of sequenced fragments multiplied by 1 million. Correction for multiple comparisons employed the Benjamini–Hochberg method for the False Discovery Rate (FDR), and data are expressed at multiple thresholds. DESeq2 involves normalization of raw read counts using median ratio normalization where the geometric mean is calculated for each transcript across all samples, and the count of a transcript in each sample is then divided by this mean. DESeq2 uses the Wald test for significance testing.

Ingenuity Pathway Analysis (Qiagen Bioinformatics, Redwood City CA) was used to group significantly affected genes into biological pathways. This analysis was conducted on the 403 transcripts differentially expressed between LND and control samples at FDR < 0.05 for both edgeR and DESeq2 methods. Output included two statistical measures. The first (p-value) represents the probability that the correlation between the genes entered did not occur by chance. The second (activation z-score) represents the degree to which observed changes correlate with the expected changes associated with pathway activation or inhibition. Significant pathway activation is reflected by a positive z-score of > 2, (at p < 0.05), while pathway inhibition is indicated by a negative z-score.

### Proteomic analyses

At least 1 million cells from each line were harvested as described above for gene expression profiles and stored at − 80 °C. Once samples from all lines were ready for simultaneous processing, they were subjected to trypsin digestion followed by LC–MS/MS of resulting peptides on an Orbitrap Fusion mass spectrometer.

All raw data files were processed together in a single run by MaxQuant (version 1.6.7) with the following settings: Group-specific parameters [Variable modifications: Oxidation(M), Acetyl (Protein N-term) and Deamidation (NQ); Digestion: trypsin]; Global parameters [Min. peptide length: 6 and Max. peptide mass [Da]: 6000, Modifications used for protein quantification: Oxidation(M), Acetyl (Protein N-term) and Deamidation (NQ)]; FTMS MS/MS match tolerance: 0.05 Da; Match between runs: true and a maximum of two missed cleavage sites were allowed. Database searches were conducted with the Andromeda search engine with the UniProt-SwissProt human canonical database as a reference and the database of common laboratory contaminants. MaxQuant reports summed intensity for each protein as well as its iBAQ value. Proteins that shared all identified peptides were combined into a single protein group. Peptides that matched multiple proteins were assigned to the protein group with the most unique peptides. MaxQuant employs the MaxLFQ algorithm for label-free quantification. Quantification was performed using unique and multiple-mapping peptides, including those modified by acetylation (N-terminal), oxidation (methionine), and deamidation (NQ). PTQX was used for general quality control^91^.

LFQ intensities of proteins generated from MaxQuant were imported into Perseus (version 1.6.7) to identify differentially expressed proteins (DEPs). Briefly, proteins identified as: only identified by site, reverse and contaminants were removed and protein group LFQ intensities were log2-transformed to reduce the effect of outliers and filtered to have 6 valid values (50% of samples). Missing LFQ values were imputed before performing Student’s t-tests. The imputation was achieved by using the “Replace missing values from normal distribution” function. Because prior studies have suggested that routine correction for multiple comparisons using the FDR method may be too conservative for discovery proteomics, differences are presented with both uncorrected p-values and FDR-corrected values. Graphic representations of the data were prepared using either R or GraphPad Prism 8.

### Functional assessment of LND lines

The functional consequences of the *HPRT1* gene variants were assessed using three approaches. The first was a direct enzyme assay for HGprt from tissue extracts, modified for cultured cells, and adapted for use in a microplate platform^92^. It is based on conversion of radiolabeled hypoxanthine into inosine monophosphate (IMP). Individual iPSC cultures were grown in 6 well dishes. Cells were detached with Accutase (Stemcell Technologies) into single cell suspensions, pelleted by centrifugation at 300×g for 4 min, and mixed with 50 µL of assay buffer consisting of 50 mM HEPES and 15 mM MgCl_2_. Samples were then frozen at − 80 °C for storage. For assay, samples were thawed, sonicated briefly, and centrifuged at 10,000×g for 10 min. A 10 µL aliquot of each was then incubated in a reaction mixture that contained 100 µM [8-^14^C]-hypoxanthine (50 mCi/mmol, Moravek Biochemicals Inc, CA), assay buffer and 10 mM PRPP (Sigma). The assay was allowed to proceed for 30 min at 37 °C. The reaction was then terminated by the addition of 5 μL of 2 M PCA. Tubes were then incubated on ice for 15 min to precipitate potassium-perchlorate. Precipitate was centrifuged and a 5 µL sample of the supernatant was spotted onto microplates with diethylaminoethyl anion exchange filters (Millipore, Billerica MA). Samples were allowed to adsorb for 60 min and free radiolabeled substrate was washed from IMP bound to the filters with 200 µL of H_2_O followed by 200 µL of 50% methanol (3 times) by vacuum filtration using a MultiScreen HTS Vacuum Manifold (Millipore). Then 100 µL of ULTIMA GOLD MV scintillation liquid (Perkin Elmer, Waltham, MA) was added and plates were stored at room temperature for 24 h before counting with a 2450 MicroBeta Microplate Scintillation Counter (Perkin Elmer, Finland). To accommodate slight changes in cell density at harvest, HGprt enzyme activity was normalized to total protein concentrations using the Pierce BCA kit (Thermo Fisher Scientific, Rockford IL).

The second approach to functional assessment of the *HPRT1* gene variants involved growth in medium supplemented with 6-thioguanine (6TG), an inert prodrug that is phosphoribosylated into a toxic nucleotide by HGprt^93^. Normal cells expressing HGprt die after 1–2 days of exposure, while HGprt-deficient cells grow normally. For each iPSC line, 50,000 cells were plated in 24-well plates in mTeSR1 media (Stemcell Technologies), with or without 30 µM 6TG (Sigma A-4660). Medium was replaced every other day. Cells survival was assessed on days 2, 4, and 6.

The third approach for functional assessment involved measuring intracellular purine levels and purine waste products released into the tissue culture medium. Cultures were grown to 80% confluency in triplicate samples (at least 1 million cells per sample). To measure purines released into culture medium, fresh medium was replaced 24 h before collecting a 450 µL aliquot of the culture medium, and mixed with 50 µL of 1 M perchloric acid and stored at − 80 °C. To measure intracellular purines, the cells were removed with Accutase and pelleted at 500×g for 5 min. Purines were measured by high performance liquid chromatography with photodiode array ultraviolet detection (HPLC–UV) as previously described^3,19,42^. This method resolves and quantifies the most abundant biologically relevant purines including ATP, ADP, AMP, adenosine, adenine, GTP, GDP, GMP, guanosine, guanine, hypoxanthine, inosine, xanthine and uric acid^17,42^. Purine analytes were normalized to total protein with the Pierce BCA kit (Thermo Fisher Scientific, Rockford, IL).

## Supplementary Information

Supplementary Information.

## Data Availability

Data or materials described in this report may be requested from the authors.
