# Supplementary material for: Induced pluripotent stem cells from subjects with Lesch-Nyhan disease
Source: Sci Rep. 2021 Apr 19;11:8523. doi: 10.1038/s41598-021-87955-9 (PMC8055678; doi:10.1038/s41598-021-87955-9)

Induced Pluripotent Stem Cells from Subjects with Lesch-Nyhan Disease

Diane J. Sutcliffe,[1*] Ashok R. Dinasarapu,[2*] Jasper E. Visser,[3,4] Joery den Hoed,[1] Fatemeh Seifar,[1,5] Piyush Joshi,[1] Irene Ceballos-Picot,[6] Tejas Sardar,[1] Ellen J. Hess,[1,5,7] Yan V. Sun,[8] Zhexing Wen,[1,9,10] Michael E. Zwick,[2,11] H. A. Jinnah[1,2,5,11. *]

1. Department of Neurology; Emory University School of Medicine, Atlanta, GA 30322
2. Department of Human Genetics; Emory University School of Medicine, Atlanta, GA 30322
3. Department of Neurology; Donders Institute for Brain, Cognition and Behavior, Radboud University Medical Center; Nijmegen, The Netherlands
4. Department of Neurology, Amphia Hospital, Breda, The Netherlands
5. Neurosciences Graduate Program, Graduate Division of Biological and Biomedical Sciences, Laney Graduate School, Emory University, 30322
6. Laboratoire de Biochimie Métabolomique et Protéomique; Hôpital Universitaire Necker
7. Department of Pharmacology and Chemical Biology; Emory University School of Medicine, Atlanta, GA 30322
8. Department of Epidemiology; Emory University Rollins School of Public Health, Atlanta, GA. 30322
9. Department of Psychiatry & Behavioral Sciences; Emory University School of Medicine, Atlanta, GA 30322
10. Department of Cell Biology; Emory University School of Medicine, Atlanta, GA 30322
11. Department of Pediatrics: Emory University School of Medicine, Atlanta, GA 30322

*Co-First Authors

***Correspondence:**
H. A. Jinnah, M.D., Ph.D.
Professor, Departments of Neurology, Human Genetics & Pediatrics
Emory University, 101 Woodruff Circle, 6305 Woodruff Memorial Building
Atlanta GA, 30322
E-mail: hjinnah@emory.edu

Supplementary Material

Supplementary Figure 1. Gene sequencing results for confirmation of LND gene variants.
Supplementary Figure 2. Karyotypes for each LND and control line.
Supplementary Figure 3. Quantitative PCR confirmation of selected findings from RNAseq.
Supplementary Figure 4. Western blot confirmation of selected findings from protemics.

Supplementary Figure 1. Gene sequencing results for confirmation of LND gene variants

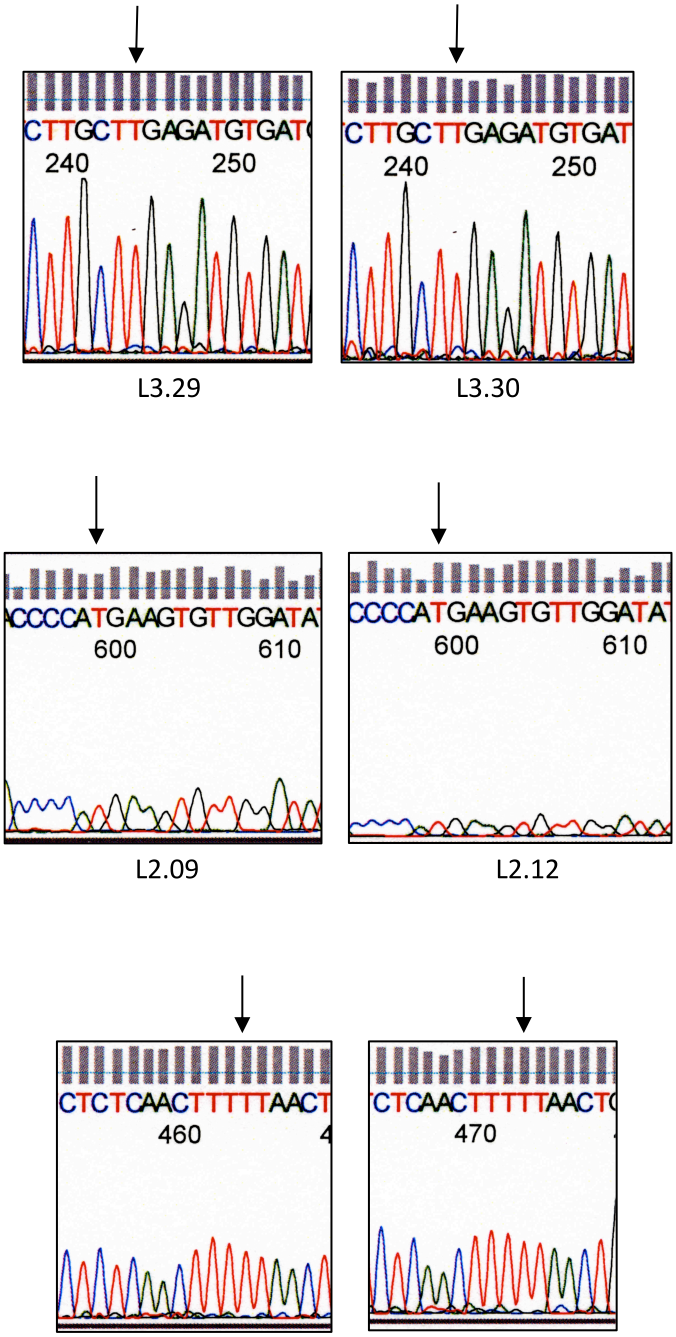

c.151C>T

L3.29 L3.30

c.508C>T

L2.09 L2.12

c.371 ins TT

L1.02 L1.04

Supplementary Figure 2. Karyotypes for each LND and control line

Control Karyotypes

C1.02

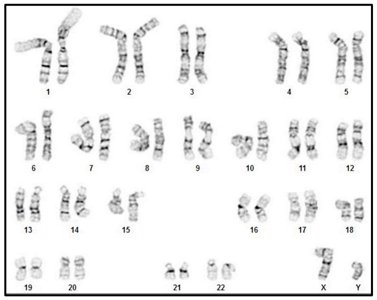

C1.03

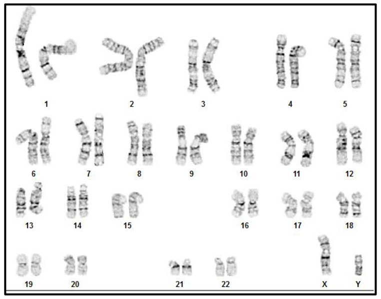

C2.04

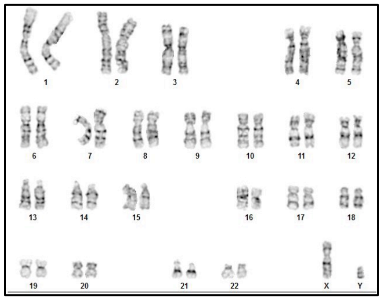

C2.06

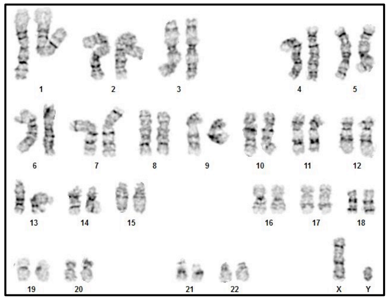

C3.05

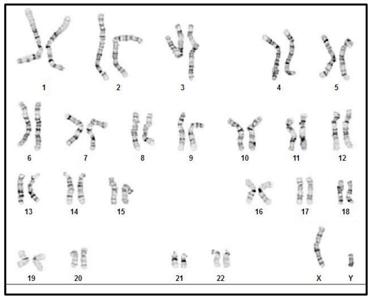

C3.08

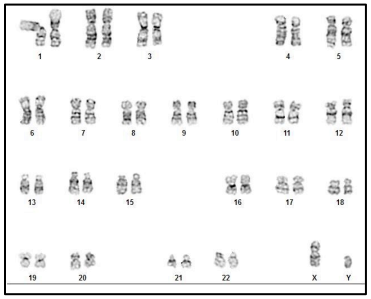

LND Karyotypes

L2.09

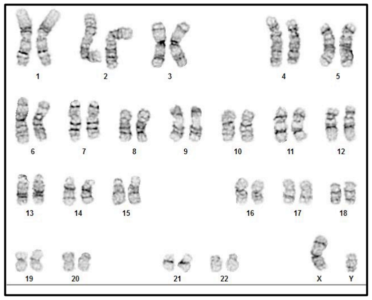

L2.12

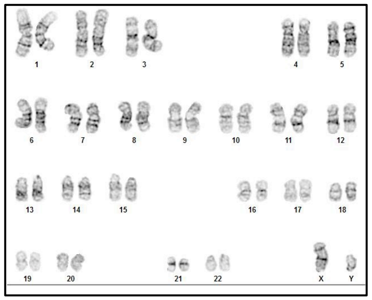

L3.29

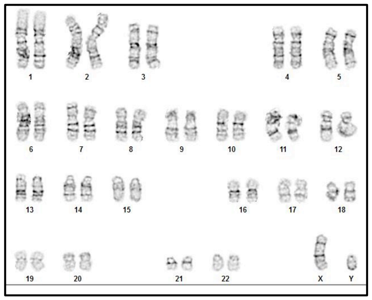

L3.30

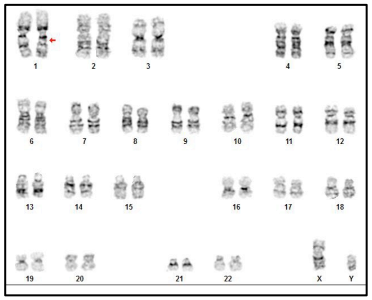

L1.02

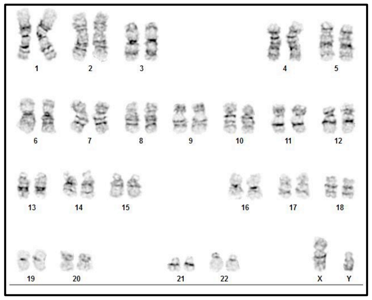

L1.04

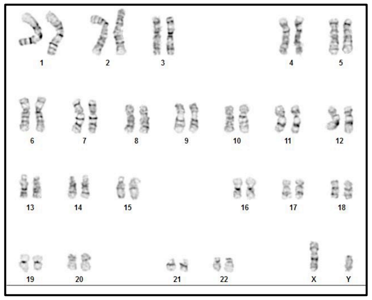

Supplementary Figure 3. Quantitative PCR confirmation of selected findings from RNAseq

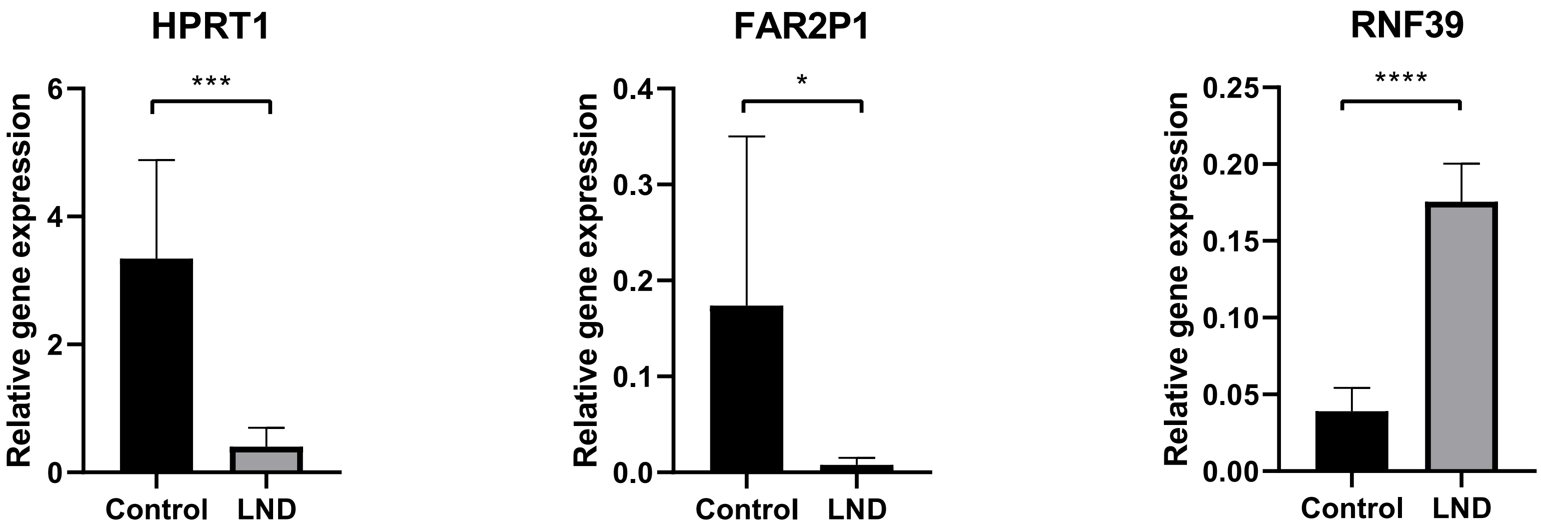

Supplementary Figure 4. Western blot confirmation of selected findings from proteomics.

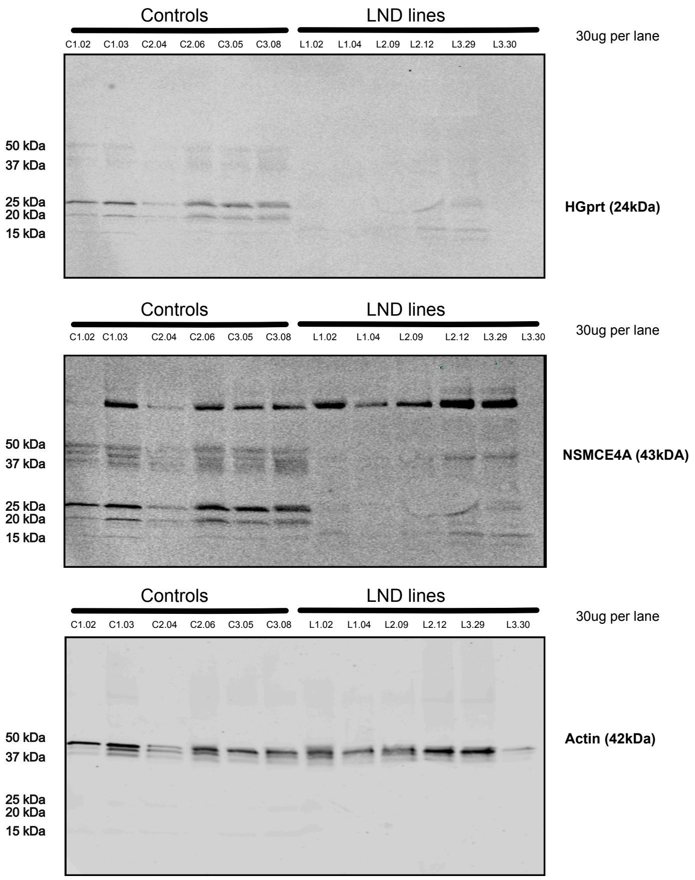

Supplement: Supplementary file 1 — Supplementary Information. [file 41598_2021_87955_MOESM1_ESM.pdf]
